# Challenges and Concerns in the Utilization of Meta-Analysis Software: Navigating the Landscape of Scientific Synthesis

**DOI:** 10.7759/cureus.53322

**Published:** 2024-01-31

**Authors:** Sankalp Yadav

**Affiliations:** 1 Medicine, Shri Madan Lal Khurana Chest Clinic, New Delhi, IND

**Keywords:** statistical models, meta regression, jasp, software, meta-analysis

## Abstract

Meta-analysis has emerged as a pivotal tool for synthesizing evidence in scientific research, facilitated by the advent of meta-analysis software. While these tools have significantly streamlined the synthesis process, challenges and concerns persist, impacting the reliability and validity of meta-analytic findings. This editorial addresses key issues in the use of meta-analysis software, including heterogeneity, publication bias, data quality, model dependence, and user competence. As the scientific community increasingly relies on meta-analytic approaches, collaborative efforts are needed to establish standardized reporting guidelines, enhance data quality, and improve transparency. This study highlights the importance of addressing these challenges to ensure the continued evolution of meta-analysis as a robust and informative method for evidence synthesis in scientific research.

## Editorial

Meta-analysis has become a cornerstone in evidence-based research, providing a systematic approach to synthesizing data from multiple studies [1]. The advent of meta-analysis software has undoubtedly enhanced the efficiency and accuracy of this process, allowing researchers to draw more robust conclusions from a wealth of information [2]. However, as the scientific community increasingly relies on these tools, several challenges and concerns have emerged, raising questions about the reliability and validity of meta-analytic findings.

Heterogeneity and inconsistency

Heterogeneity in meta-analysis pertains to the extent of variation among the findings of distinct studies. The primary presumption for conducting a meta-analysis is that the populations, interventions, controls, and outcomes of the studies are all identical. One crucial step in performing a meta-analysis is evaluating the heterogeneity among the primary studies. If there is a sizeable amount of heterogeneity, the study should concentrate on identifying and comprehending the sources of the variation. Meta-analysis assesses whether primary studies are heterogeneous and evaluates the variation in their findings [2]. There are a number of strategies to address the issues of heterogeneity, including checking again if the data are correct, omitting meta-analysis where there exists a substantial variation in results and, especially when there is inconsistency in the direction of effect, exploring the heterogeneity by conducting subgroup analyses or meta-regression, ignoring heterogeneity when doing fixed-effect meta-analyses, performing a random-effects meta-analysis when heterogeneity cannot be explained, changing the effect measure, or excluding one or two studies with results that conflict with the majority of the studies [3].

One of the primary challenges in meta-analysis software is the management of heterogeneity among studies. The inclusion of diverse study designs, populations, and methodologies can lead to substantial variability, potentially influencing the overall effect size. Current software tools often employ statistical methods to address heterogeneity, but the complexity of real-world data poses difficulties in achieving complete homogeneity, especially when heterogeneity is unexplainable or in data where few studies have significant differences in results from the majority.

Publication bias and selective reporting

Meta-analyses are susceptible to publication bias, where studies with statistically significant results are more likely to be published than those with non-significant findings [2]. Some software tools, like Comprehensive Meta-Analysis (Canton, OH: Biostat, Inc.), attempt to address this bias through funnel plot asymmetry tests, but the effectiveness of these methods remains a subject of debate as these funnel-plot-based tests are often underpowered and allow for multiple interpretations. Other software, like MedCalc (Ostend, Belgium: MedCalc Software Ltd.), uses Egger's and Begg's tests to reduce publication bias. Moreover, the challenges posed by selective reporting of outcomes in individual studies add another layer of complexity to the synthesis process [4].

Data quality and standardization

The quality of meta-analytic results heavily relies on the quality of the input data. Variations in data collection methods, reporting standards, and measurement tools across studies can introduce inconsistencies. Meta-analysis often involves combining data from studies with varying methodologies, study designs, and data collection techniques. Heterogeneity in data sources can introduce challenges in ensuring the quality and compatibility of the data, potentially leading to biased or unreliable results. Studies included in meta-analyses may suffer from incomplete or inaccurate reporting, making it challenging for researchers to extract relevant information. Missing data or discrepancies in reported results can compromise the overall quality of the meta-analysis. The manual input of data into meta-analysis software leaves room for data entry errors. Even minor inaccuracies in the input data can have a cascading effect, influencing effect sizes and potentially leading to erroneous conclusions [2].

Studies included in meta-analyses may use diverse measurement scales and units, posing challenges in standardizing data for meaningful comparisons. The absence of standardized units can hinder the integration of findings and may require additional statistical adjustments. Meta-analysis software may encounter challenges in handling data with different formats, structures, or coding schemes. Inconsistencies in data format across studies can impede the seamless integration of information and necessitate additional preprocessing efforts.

Meta-analysis software should incorporate tools for assessing the quality of the included studies. Quality assessment criteria can help researchers identify potential biases and ensure that only reliable data contribute to the overall analysis. Implementation of robust data cleaning and validation protocols within meta-analysis software is crucial. Automated checks for data consistency, range validation, and identification of outliers can help minimize data entry errors and enhance overall data quality. Meta-analysis software should integrate algorithms for standardizing diverse data, including converting measurement scales and units. The implementation of standardization protocols ensures that data from different sources can be harmonized for meaningful synthesis. While some software packages provide tools for data standardization, the lack of universally accepted standards poses difficulties in achieving seamless integration and comparability of diverse datasets.

Assumptions and model dependence

Meta-analysis software often relies on specific statistical models, and the results are contingent upon the validity of underlying assumptions. The validity of the within-study normality assumption could be affected by multiple factors, such as individual studies’ sample size, event probabilities of binary outcomes, and true distributions of continuous measures. Deviations from these assumptions, such as violations of the independence assumption or normality of residuals, can compromise the reliability of the findings [5].

The choice of meta-analysis software introduces an additional layer of complexity, as different programs may employ distinct statistical models, algorithms, and underlying assumptions. Model dependence refers to the sensitivity of results to the specific statistical model chosen for analysis. Researchers must recognize that different software packages may yield divergent results for the same dataset, emphasizing the importance of model robustness and sensitivity analyses. When there is a correlation between two or more studies under evaluation due to shared data or researchers, the dependent structures in a meta-analysis may prove helpful. Researchers need to be aware of the limitations associated with different models and the potential impact on the robustness of the meta-analysis.

The developers of meta-analysis software play a pivotal role in advancing the field. They should prioritize incorporating user-friendly interfaces, providing clear documentation on underlying assumptions, and offering options for sensitivity analyses. Additionally, collaboration between statisticians, software developers, and domain experts is essential to ensure that software tools remain adaptable to evolving research methodologies and can accommodate the complexities of diverse datasets.

User competence and interpretation

The proficiency of researchers in using meta-analysis software plays a pivotal role in the accuracy of the results. Inadequate understanding of statistical concepts, misinterpretation of software outputs, and lack of transparency in reporting can lead to erroneous conclusions. Meta-analysis software often requires a solid foundation in statistics and research methodology. Insufficient statistical literacy among users may lead to misinterpretation of results, improper application of analysis techniques, and an increased risk of methodological errors. Some meta-analysis software tools may have complex interfaces that require users to navigate through various settings and options. Inadequate training or familiarity with software functionalities may hinder researchers from making optimal choices during the analysis process. In multidisciplinary research, collaboration between experts with diverse backgrounds is common. User competence challenges may arise when researchers from different fields attempt to use meta-analysis software without a shared understanding of statistical principles and methodologies. Users may misinterpret effect sizes and confidence intervals, leading to incorrect conclusions about the magnitude and precision of the synthesized effect. A lack of understanding of these statistical concepts can compromise the validity of meta-analytical findings. Overlooking heterogeneity and sensitivity analyses and ignoring assumptions and limitations could prove decisive at the time of the results.

There is a need for comprehensive training and guidelines to enhance user competence and promote standardized reporting practices. Besides, software developers should prioritize user-friendly interfaces with clear instructions and tooltips to guide users through the analysis process. Intuitive designs can reduce the learning curve and enhance the accessibility of meta-analysis software. Some commonly used meta-analysis software and issues related to them are detailed in Table 1 [6-8].

**Table 1:** Some commonly used meta-analysis software and issues related to them.

Software	Strengths	Weakness
Review Manager (RevMan) (London, UK: Cochrane Collaboration)	User-friendly interfaces, risk of bias assessment tool, and plenty of support available	Limited statistical functions and graphic customization capabilities, initial software setup is complicated and the software must be downloaded separately
MetaXL (Queensland, Australia: EpiGear International Pty Ltd.)	User-friendly interfaces, can produce Doi plots showing normal-quantile against effect size	Limited statistical functions and graphic customization capabilities, initial software setup is complicated and the software must be downloaded separately
Stata (College Station, TX: Stata Corporation)	User-friendly interface, good data visualization, and easy download in multiple formats are available	Paid software, struggles when small-study effects emerge, and outcome data is unavailable
R (Vienna, Austria: R Foundation)	Free to use, outstanding features for customizing graphics and statistics	Demands programming knowledge from users, which reduces the user base
MetaGenyo (Granada, Spain: Genyo)	User-friendly, used to perform meta-analyses on genetic association studies	Mainly used for genetic association studies
Meta-Analysis	Free to use	Not updated
Comprehensive Meta-Analysis (Canton, OH: Biostat, Inc.)	Allow users to enter effect sizes of different formats and the comprehensiveness of the numerical options and output	Direct import of text or other data files is not plausible and lacks the American Psychological Association standard output feature
WEasyMA 2.5 (ClinInfo: Lyon, France)	Fast in its speed	Data cannot be imported or pasted
MIX 1.5 (Salinas, CA: BiostatXL)	Extensive graphical output, detailed numerical options, built-in data sets, and extensive tutor functions	Requires Microsoft Excel to run, limitation on the maximum number of data sets
MetaStat (Natick, MA: MetaStat, Inc.)	Effect size calculator	Not the most elegant user interface
OpenMeta[Analyst] (Providence, RI: Brown University)	User-friendly interface, execute meta-regression	Isolated reports of frequent crashes
MetaWin 2.1 (Richmond, VA: Rosenberg et al.)	Effect size calculator, option to use bootstrap confidence intervals	Help files and the comprehensive manual are extensive
Open Meta-analyst for Ecology and Evolution (OpenMEE) (Providence, RI: Brown University)	Centered on the ecology and evolutionary field, execute meta-regression	Runs via R programs
MetaEasy (Excel add-on) (Kontopantelis and Reeves)	Works in Excel	Limited functionality
Meta-Essentials (Excel workbook) (Rotterdam, Netherlands: ERIM)	User-friendly interface	Lacks American Psychological Association standard output feature
Jamovi (Love et al.)	Free to use, provides outputs in American Psychological Association standard	R knowledge may be needed for higher functionalities
PythonMeta package (Bern, Switzerland: University of Bern)	Combining effect measures, subgroup analysis, cumulative meta-analysis, sensitivity analysis (one or two factors), plots drawing: forest plot, funnel plot, bar-line, and cross-block plot	Lacks features like multivariate analysis
Anaconda (Austin, TX: Anaconda, Inc.)	Multiple environments, easy package installation, packages installed in multiple environments are hard-linked	It comes with numerous packages by default, occupying a lot of space, Conda package manager is fragile and slow
Jeffreys's Amazing Statistics Program (JASP) (Amsterdam, Netherlands: The JASP Team)	User-friendly interface, emphasizes Bayesian and classical techniques, open source	In comparison to older software, it has fewer sophisticated statistical techniques, no command-line interface, and is less well-known in the academic community

Meta-analysis software plays a pivotal role in addressing challenges associated with meta-regression and network meta-analysis. The complexity of regression models in meta-analysis software can be mitigated through the integration of user-friendly interfaces that guide researchers in applying regularization techniques and conducting thorough diagnostic checks. Moreover, advanced software features, such as imputation algorithms, can aid in handling missing data, ensuring the completeness and quality of datasets for meta-regression analyses.

Similarly, in the context of network meta-analysis, sophisticated meta-analysis software should offer tools for assessing consistency and managing data. The software can facilitate sensitivity analyses, allowing researchers to explore the impact of different modeling assumptions and priors, thereby enhancing the robustness of network meta-analysis results. Additionally, incorporating expert input directly into the software, perhaps through collaborative platforms, can assist in addressing the subjective nature of choosing prior distributions and contribute to the credibility of the synthesized evidence. By addressing these issues within the framework of meta-analysis software, researchers can leverage these powerful methodologies effectively for more informed decision-making in various fields.

The utilization of meta-analysis software has undoubtedly transformed the landscape of evidence synthesis, but challenges persist. Addressing these concerns requires a concerted effort from researchers, software developers, and the scientific community at large. Collaborative initiatives to establish standardized reporting guidelines, enhance data quality, and improve transparency in methodologies will contribute to the continued evolution of meta-analysis as a reliable and informative tool in scientific research. As we navigate the complex terrain of meta-analysis software, a commitment to rigorous methodology, critical evaluation, and ongoing refinement is essential to ensure the integrity and credibility of synthesized scientific knowledge.
